# Do Corticosteroid Receptor mRNA Levels Predict the Expression of Their Target Genes?

**DOI:** 10.1210/jendso/bvac188

**Published:** 2022-12-12

**Authors:** Lisa L Koorneef, Eva M G Viho, Lucas F Wahl, Onno C Meijer

**Affiliations:** Department of Internal Medicine, Division of Endocrinology, Leiden University Medical Center, Leiden 2333 ZA, the Netherlands; Einthoven Laboratory for Experimental Vascular Medicine, Leiden University Medical Center, Leiden 2333 ZA, the Netherlands; Department of Internal Medicine, Division of Endocrinology, Leiden University Medical Center, Leiden 2333 ZA, the Netherlands; Einthoven Laboratory for Experimental Vascular Medicine, Leiden University Medical Center, Leiden 2333 ZA, the Netherlands; Department of Internal Medicine, Division of Endocrinology, Leiden University Medical Center, Leiden 2333 ZA, the Netherlands; Einthoven Laboratory for Experimental Vascular Medicine, Leiden University Medical Center, Leiden 2333 ZA, the Netherlands; Department of Internal Medicine, Division of Endocrinology, Leiden University Medical Center, Leiden 2333 ZA, the Netherlands; Einthoven Laboratory for Experimental Vascular Medicine, Leiden University Medical Center, Leiden 2333 ZA, the Netherlands

**Keywords:** glucocorticoid receptor, mineralocorticoid receptor, glucocorticoid-induced leucine zipper, hippocampus, gene expression, single-cell RNA sequencing

## Abstract

The glucocorticoid stress hormones affect brain function via high-affinity mineralocorticoid receptors (MRs) and lower-affinity glucocorticoid receptors (GRs). MR and GR not only differ in affinity for ligands, but also have distinct, sometimes opposite, actions on neuronal excitability and other cellular and higher-order parameters related to cerebral function. GR and MR messenger RNA (mRNA) levels are often used as a proxy for the responsiveness to glucocorticoids, assuming proportionality between mRNA and protein levels. This may be especially relevant for the MR, which because of its high affinity is already largely occupied at low basal (trough) hormone levels. Here we explore how GR and MR mRNA levels are associated with the expression of a shared target gene, glucocorticoid-induced leucine zipper (GILZ, coded by *Tsc22d3*) with basal and elevated levels of corticosterone in male mice, using in situ hybridization. Depending on the hippocampal subfield and the corticosterone levels, mRNA levels of MR rather than GR mostly correlated with GILZ mRNA in the hippocampus and hypothalamus at the bulk tissue level. At the individual cell level, these correlations were much weaker. Using publicly available single-cell RNA sequencing data, we again observed that MR and GR mRNA levels were only weakly correlated with target gene expression in glutamatergic and GABAergic neurons. We conclude that MR mRNA levels can be limiting for receptor action, but many other cell-specific and region-specific factors ultimately determine corticosteroid receptor action. Altogether, our results argue for caution while interpreting the consequences of changed receptor expression for the response to glucocorticoids.

Glucocorticoids have extensive effects on emotional and cognitive processes in the brain, which allows for adaptation to stressors. Glucocorticoids bind to the high-affinity mineralocorticoid receptors (MRs, coded by *Nr3c2*) and lower-affinity glucocorticoid receptors (GRs, coded by *Nr3c1*), which each coordinate distinct processes [1]. MR and GR act in large measure as ligand-activated transcription factors. They have a very similar central DNA binding domain, via which they bind to accessible glucocorticoid response elements (GREs) at the genome. Although MRs and GRs share downstream target genes, they also each regulate specific target genes based on additional interactions at the DNA [2, 3]. GRs can additionally repress gene transcription via “negative GREs” or in conjunction with other transcription factors such as AP-1 and nuclear factor κB [4-6].

In the hippocampus, where MR has its highest expression, GRs and MRs are mainly known to functionally oppose each other, but synergistic actions have also been described [1, 7-9]. At the cellular level (eg, neuronal excitability), these opposite actions translate into a U-shaped dose-dependent response to corticosterone [7, 10, 11]. In these cases, the hormone effects depend on the balance between GR- and MR-mediated actions. At the organism level, this has led to the theory that a proper GR:MR balance is essential for behavioral adaptation and neuroendocrine functioning [12, 13]. In theory, a combination of transactivation at low steroid levels via MR, followed by transrepression via GR at higher steroid levels, would allow for such a U-shaped transcriptional response. Alternatively, a differential transcriptional response may also arise from the 10-fold lower maximum level of transactivation by MRs compared to GRs [14]. Regardless of the directionality of the MR- and GR-mediated effects, receptor expression levels are thought to be important for hormone responsiveness [15].

Because glucocorticoid effects on the brain are crucial for stress adaptation and mental health, many studies assess steroid responsiveness by measurement of MR and GR messenger RNA (mRNA) levels, assuming proportionality between mRNA and protein levels [16-20]. Particularly for MR this may be relevant, as its high affinity leads to substantial ligand occupancy even at basal (trough) levels of hormone. Regulation of receptor abundance would then be a way to change the strength of what is a tonic signal via MR [21, 22]. The lower affinity of GRs makes corticosteroid hormone concentration a first limiting step. However, once GRs get activated, receptor number may be limiting for particular transcriptional and cellular responses.

In this study we explore in the mouse brain how GR and MR mRNA levels, and the GR:MR balance at the mRNA level, are correlated with the expression of shared target gene glucocorticoid-induced leucine zipper (GILZ, coded by *Tsc22d3*). This association was investigated both at the tissue and cellular levels in the hippocampus and paraventricular nucleus of the hypothalamus (PVN) of mice given a vehicle or corticosterone injection before being humanely killed. We chose the hippocampus and the PVN as our regions of interest because GR and MR are both expressed and functionally important in these brain structures [1, 23]. GR, MR, and GILZ mRNA levels were measured with in situ hybridization (ISH), as ISH is one of the few quantitative methods with spatial resolution. To further distinguish between neuronal cell types, the relation between corticosteroid receptors and target gene expression was also analyzed using the published Allen Brain Atlas single-cell RNA sequencing data set of the mouse hippocampus.

## Materials and Methods

### Animals

This study was approved by the institutional ethics committee on animal care and experimentation at the Leiden University Medical Center. Eight-week-old male C57Bl/6J mice (Charles River) were housed in ventilated cages with a 12-hour light:12-hour darkness regimen and ad libitum access to food and water. Mice were injected subcutaneously with 200 μL solvent (EtOH:PBS 1:20) or 1.5 mg/kg corticosterone (Sigma-Aldrich) 1 hour before being humanely killed by cervical dislocation followed by rapid decapitation in a separate room (n = 8 mice/group; 16 mice in total) [24]. Brains were collected and stored at −80 ° for ISH.

### In Situ Hybridization

Frozen, unfixed brain slices (12 µm) were collected on glass slides at the bregma −0.772 mm (PVN) and −1.532 mm (hippocampus). Four-plex ISH was performed using the RNAScope fluorescent multiplex assay and RNAscope 4-plex ancillary kit (Advanced Cell Diagnostics) and opal fluorophores 520, 570, 620, and 690 (PerkinElmer). Nuclei were counterstained with 4', 6-diamidino-2-phenylindole (DAPI) staining (Advanced Cell Diagnostics). Results of the FKBP5 heterogeneous nuclear RNA probe that we included were later excluded from the analysis, as the signal was too low. Pictures were captured with a confocal microscope (SP8 WLL, Leica Microsystems). Images were visually inspected for the quality of the staining and were excluded if the overall signal intensity across all channels deviated substantially from the mean intensity, which was otherwise fairly constant throughout the experiment.

### Image Analysis

All pictures were analyzed in 8-bit with ImageJ Software (US National Institutes of Health). Cells were selected based on nuclear DAPI staining as follows. First, gaussian blur was used to autofill nuclei and the contrast of all DAPI images was enhanced. Nuclei were then selected by a fixed threshold and the outer edge was enlarged by a fixed size: 5 pixels for CA1, CA3, and PVN and 0.5 for the nucleus-dense dentate gyrus (DG). Touching nuclei were separated with the watershed function, after which nuclei were selected using the “analyze particles” function. Quality of selections were manually verified and overlapping cells were deleted from the analysis. The signal of each probe was then measured within these cell selections with automatic thresholding. The “otsu” method [25] was used for the quantification of the MR signal in all regions and of the GR and GILZ signal in CA1, CA3, and PVN, while the “moments” method [26] better identified the GR and GILZ signal in the DG because of the higher background signal in this region. Expression was defined as the percentage area covered by the signal compared to the total cell area. Cells were classified as GILZ positive if the percentage area was greater than 0 in the CA3, CA1, and PVN, and greater than 3 in the DG. To display the cell distribution per brain region and treatment, cells were analyzed with the single-cell clustering package Seurat v.3.1.5 in R v4.0.0 [27]. The same package was used to determine the number of genes expressed per cell, as well as the distribution of MR, GR, and GILZ mRNA expression per cell and per mouse [27].

### Single-cell RNA Sequencing Data Resources, Metrics, and Processing

Single-cell data were obtained from the SMART-seq single-cell RNA sequencing data set published by the Allen Institute for Brain Science [28]. The gene expression matrix and the table of cell metadata were downloaded from https://portal.brain-map.org/atlases-and-data/rnaseq/mouse-whole-cortex-and-hippocampus-smart-seq. The metadata were used to subset cells of the hippocampal formation from the gene expression matrix. We selected 9 subclasses of hippocampal cells according to the Common Cell Type Nomenclature guidelines, including 5 GABAergic neuronal cell types and 4 glutamatergic neuronal cell types [29]. The gene expression matrix from the hippocampus contained 6474 cells for 39 236 genes and was processed using the Seurat v.3.1.5 pipeline as previously described [27]. The quality control metrics showed that most cells expressed between 2500 and 13 000 genes and most genes had less than 1.00e+06 RNA counts. All cells or genes that did not meet those criteria were excluded for further analysis. The filtered data set was then log-normalized and scaled according to standard single-cell RNA sequencing data processing [27]. We performed principal component analysis and selected the top 50 principal components for clustering and t-distributed stochastic neighbor embedding (t-SNE) dimensional reduction and identification of the different cell types. The transcriptomic data within these cell types were then analyzed and displayed as scatterplots with linear regression using ggplot2 tools in R v4.0.0.

### Statistical Analysis

Simple linear regression and *t* tests were performed with GraphPad Prism (version 8.1.1.330, GraphPad Software). Stepwise multiple linear regression was performed in SPSS Statistics (version 25.0, IBM Corporation) and single-cell RNA sequencing data were analyzed in R v4.0.0. All data are expressed as mean ± SEM. All *P* values are 2-tailed and *P* less than .05 was considered statistically significant. Two groups having equal SD were analyzed with an independent-sample *t* test. Two groups having different SD were analyzed with a Welch *t* test. To calculate significance of correlates at the tissue level, a stepwise multiple linear regression was performed with GILZ mRNA expression as the dependent variable, and GR mRNA expression, MR mRNA expression, and GR:MR ratio as independent variables. Significance of correlates at the single-cell level was calculated with simple linear regression. In all regression analyses the pattern of residuals was random, supporting the linear fit of the model. The Pearson correlation coefficient was calculated to determine the strength of relationships.

## Results

### Corticosterone Induced Glucocorticoid-induced Leucine Zipper Messenger RNA Expression in the Paraventricular Nucleus of the Hypothalamus, but Not in Hippocampal Regions

**Figure 1.**
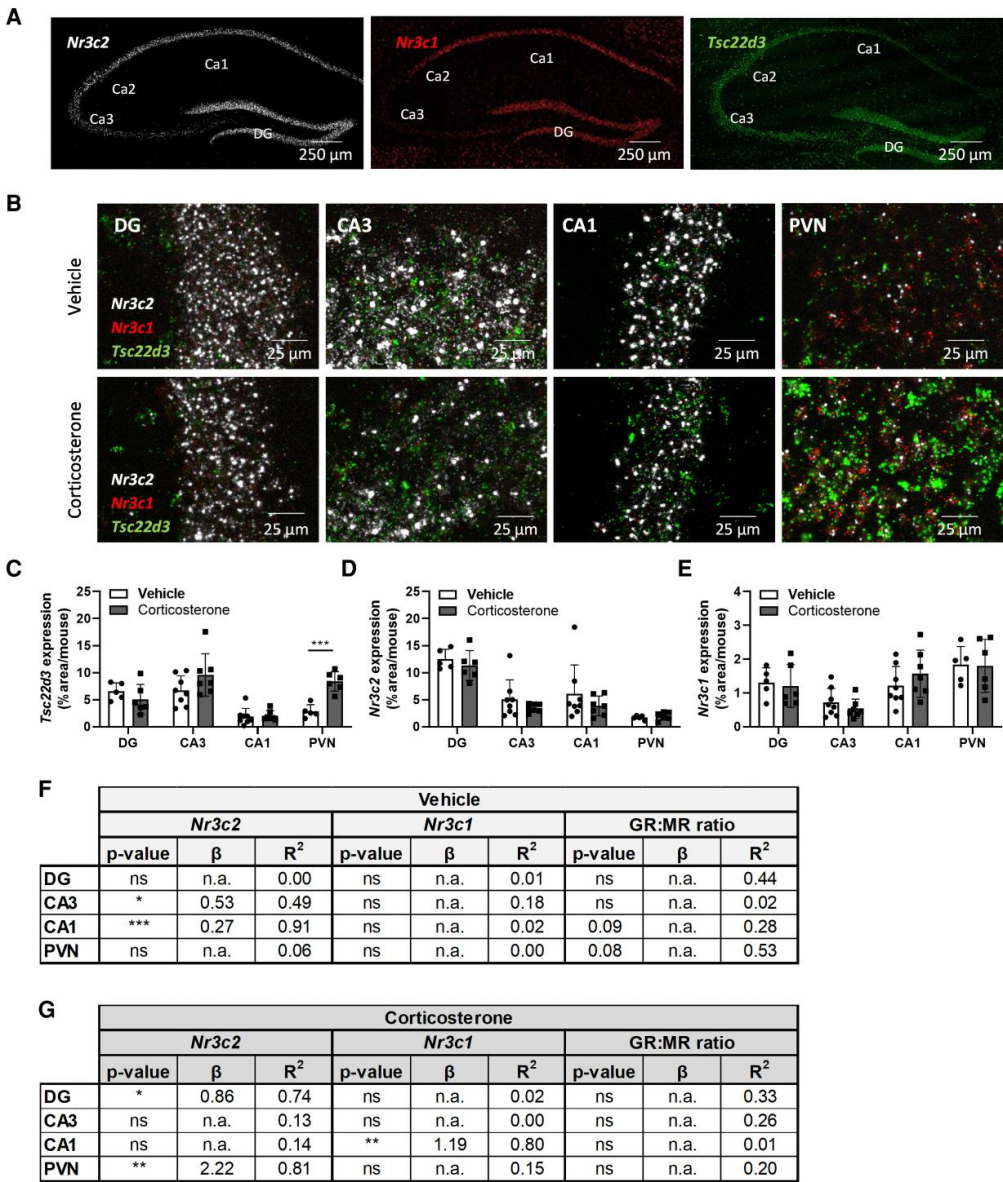
Mineralocorticoid receptor (MR) messenger RNA (mRNA) levels predict glucocorticoid-induced leucine zipper (GILZ) mRNA expression in subfields of the hippocampus and paraventricular nucleus of the hypothalamus (PVN) under basal or corticosterone conditions. Mice received a vehicle or corticosterone injection 1 hour before being humanely killed. In situ hybridization was performed to visualize MR (*Nrc3c2,* left panel), GR (*Nrc3c1*, middle panel) and GILZ (*Tsc22d3*, right panel) mRNA in hippocampal dentate gyrus (DG), CA3, and CA1 and in PVN. A, Representative images of the hippocampus of vehicle-treated animals. B, Higher magnifications with the dots representing individual mRNA molecules. C to E, Mean expression of *Tsc22d3*, *Nr3c2*, and *Nr3c1* per mouse. Corticosterone only induced *Tsc22d3* expression in PVN as calculated with an independent sample *t* test. F and G, Stepwise multiple linear regression data to assess if glucocorticoid receptor (GR) and MR mRNA levels and GR:MR ratio predicted GILZ mRNA expression. The tables show the statistical significance (*P*), slope (β), and strength (*R*^2^) of the correlations found. Values are means ± SEM of n = 5-8 animals. *P less than .05, **P less than .01, ***P less than .001.

Cellular GR (*Nr3c1*), MR (*Nr3c2*), and GILZ (*Tsc22d3*) mRNA levels were quantified with ISH on brains of mice that were pretreated with vehicle or corticosterone 1 hour before death. Visualization of the whole hippocampus and PVN showed that MR and GILZ mRNAs were highly expressed in these regions under vehicle conditions (Fig. 1A). GR mRNA levels were high in the PVN, CA1, and DG, but lower in the CA3, in concordance with many previous studies [30] (see Fig. 1A). At a higher magnification the individual dots, representing single mRNA molecules [31], could be identified for each probe and these images were used for all image quantifications (Fig. 1B). To quantify the expression of each gene per cell, cells were identified by enlarging the outer edge of nuclei that had been selected by DAPI staining. The effect of corticosterone treatment was determined by calculating the mean mRNA expression of GILZ, MR, and GR in all cells within a region per mouse. Corticosterone induced GILZ mRNA expression significantly in the PVN, but not in the DG, CA3, and CA1 (Fig. 1B and 1C). Corticosterone did not significantly affect MR and GR mRNA levels in any of the regions examined (Fig. 1B, 1D, and 1E).

### Averaged Across Mice, Mineralocorticoid Receptor Messenger RNA (mRNA) Levels Predict Glucocorticoid-induced Leucine Zipper mRNA Expression in Subfields of the Hippocampus and Paraventricular Nucleus of the Hypothalamus Under Basal or Corticosterone Conditions

To investigate if GR and MR mRNA levels and GR:MR ratio predict GILZ mRNA expression, a stepwise multiple linear regression was performed on the mean values per mouse (Fig. 1F and 1G), and for visualization purposes also a single regression for all variables separately (Supplementary Fig. S1 [32]). MR mRNA levels strongly correlated with GILZ mRNA in the DG of corticosterone-treated, but not vehicle-treated mice (*R*^2^ = 0.74; see Fig. 1F and 1G, Supplementary Fig. S1A-S1C [32]). No significant correlation was found between GR and GILZ mRNA in this region (see Fig. 1F and 1G, Supplementary Fig. S1A-S1C [32]). A nonsignificant, inverse correlation was found between GR:MR ratio and GILZ mRNA expression, likely simply reflecting the positive correlation of GILZ and MR mRNA. (see Fig. 1F and 1G, Supplementary Fig. S1C [32]). In the CA3 and CA1, MR mRNA levels predicted GILZ mRNA expression in vehicle-treated animals (*R*^2^ = 0.49 and 0.91, respectively; see Fig. 1F, Supplementary Fig. S1D and S1G [32]). In the CA1, GR mRNA levels were also strongly correlated with GILZ mRNA in corticosterone-treated animals (*R*^2^ = 0.80; see Fig. 1G, Supplementary Fig. S1H [32]). The GR:MR ratio was not strongly associated with GILZ mRNA in this region (see Fig. 1F and 1G, Supplementary Fig. S1F [32]). In the PVN, MR mRNA levels significantly correlated with GILZ mRNA expression in corticosterone-treated animals (*R*^2^ = 0.81; see Fig. 1G, Supplementary Fig. S1J [32]), but not under basal conditions (see Fig. 1F, Supplementary Fig. S1J [32]). In conclusion, MR mRNA levels predicted GILZ mRNA expression under basal conditions in the CA3 and CA1, and under corticosterone conditions in the DG and PVN, whereas GR mRNA levels predicted GILZ mRNA only in the CA1 hippocampal region of corticosterone-treated animals. GR:MR ratio was not an independent determinant for *GILZ* gene regulation and was therefore excluded from further analyses.

### Cellular Mineralocorticoid Receptor, Glucocorticoid Receptor, or Glucocorticoid-induced Leucine Zipper Messenger RNA Levels Vary Greatly Within and Between Animals

**Figure 2.**
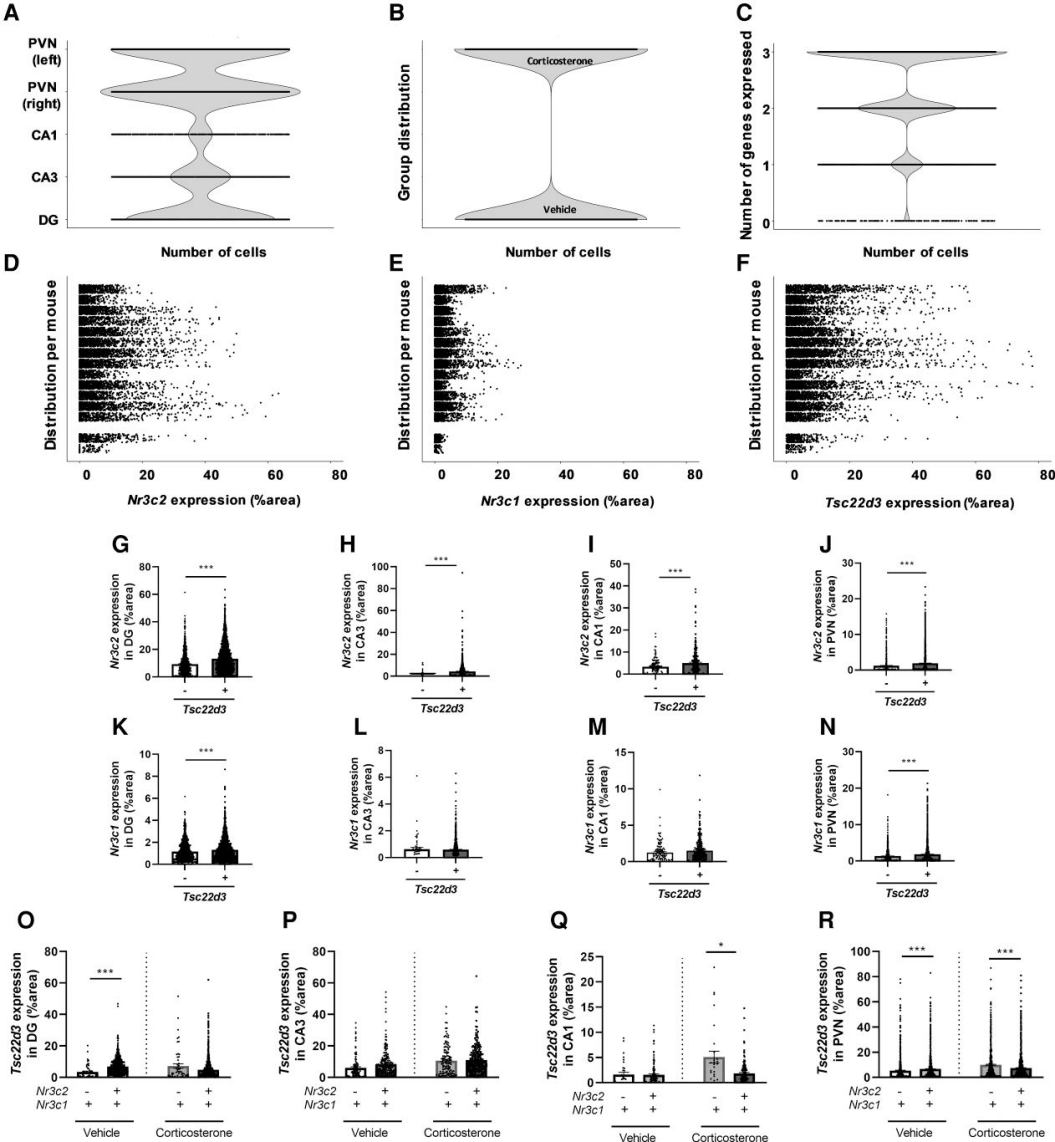
Mineralocorticoid receptor (MR) messenger RNA (mRNA) levels are higher in glucocorticoid-induced leucine zipper (GILZ)-positive cells in the hippocampus and paraventricular nucleus of the hypothalamus (PVN). A, PVN and dentate gyrus (DG) were the most nucleus-dense brain regions. B, The analysis represented an equal number of cells from vehicle- and corticosterone-treated mice. C, Most cells expressed all 3 genes, that is, *Nr3c2*, *Nr3c1*, and *Tsc22d3*, but there were also cell populations expressing only 1 or 2 of these genes. D to F, Expression of *Nr3c2*, *Nr3c1*, and *Tsc22d3* varied greatly within and between animals. Each row represents the gene expression data of one animal. G to J, *Nrc3c2* expression was higher in GILZ-positive than in GILZ-negative cells in all investigated regions. K to N, *Nrc3c1* expression was higher in GILZ-positive cells in DG and PVN, but not in CA1 and CA3. O to R, The presence of MR in glucocorticoid receptor (GR)-positive cells corresponded with lower GILZ mRNA levels under corticosterone conditions significantly in CA1 and PVN, nonsignificantly in DG, but not in CA3. Values are means ± SEM of n = 5-8 animals. Statistical significance was calculated with a Welch *t* test. .*P less than .05, ***P less than .001.

To explore the relationship between receptor levels and GILZ mRNA expression at the cellular level, we first assessed the distribution and variability of the data set. Most of the cells were detected in the PVN and DG, while the CA1 contained the lowest number of cells (Fig. 2A). The number of cells in each treatment group for all areas was comparable when cells from all regions were pooled (Fig. 2B). The majority of cells expressed both corticosteroid receptors and GILZ, and very few cells expressed none (or undetectable levels) of the 3 genes of interest (Fig. 2C). When clustered per mouse, it was apparent that the cellular expression of MR, GR, or GILZ mRNA was highly variable within and between animals (Fig. 2D-2F). Of the 2 mice with the lowest total cell number, DG cells were excluded from analysis because of technical issues (see Fig. 2D-2F).

### Mineralocorticoid Receptor Messenger RNA Levels are Higher in Glucocorticoid-induced Leucine Zipper–positive Cells in the Hippocampus and Paraventricular Nucleus of the Hypothalamus

To evaluate the enrichment of corticosteroid receptors in GILZ-responsive cells in the hippocampus and PVN, we measured MR and GR mRNA expression in GILZ*-*negative and -positive cells per region in mice from both treatment groups. MR mRNA levels were higher in GILZ*-*positive cells than in GILZ*-*negative cells in all regions of interest (Fig. 2G-2J). However, GR mRNA levels were higher in GILZ*-*positive cells only in the DG and the PVN (Fig. 2K-2N). The higher expression of the GILZ target gene in the presence of corticosteroid receptors may in itself indicate a role of the receptors in regulating GILZ in the hippocampus and PVN.

### Presence of Mineralocorticoid Receptor in Glucocorticoid Receptor–Positive Cells Is Associated With Lower Glucocorticoid-induced Leucine Zipper Messenger RNA Levels Under Corticosterone Conditions in the Hippocampus and Paraventricular Nucleus of the Hypothalamus

Recently, it has been shown that MRs may fine-tune hippocampal MR:GR balance by decreasing GR sensitivity to glucocorticoids via FKBP5 [22]. Therefore, we hypothesized that cells expressing GR but not MR may have an enhanced transcriptional response after glucocorticoid treatment. To test this, we measured GILZ mRNA levels in GR-positive cells with and without coexpressed MR, and stratified them for corticosterone treatment. Under corticosterone conditions, the presence of MR in GR-positive cells was significantly associated with decreased GILZ mRNA levels in CA1 (Fig. 2Q) and PVN (Fig. 2R), and nonsignificantly in the DG (*P* = .11; Fig. 2O) but not in CA3 (Fig. 2P). Interestingly, under vehicle conditions *Tsc22d3* expression was higher in cells coexpressing MR and GR than in cells that were positive only for GR in DG and PVN (see Fig. 2O and 2R). Together this suggests that the individual actions of MR and GR, as well as the interplay between the 2 receptors, ultimately determine *Tsc22d3* expression in the hippocampus and PVN.

### Mineralocorticoid Receptor and Glucocorticoid Receptor Messenger RNA (mRNA) Levels Weakly Predict Glucocorticoid-induced Leucine Zipper mRNA Expression at the Cellular Level in the Hippocampus and Paraventricular Nucleus of the Hypothalamus

**Figure 3.**
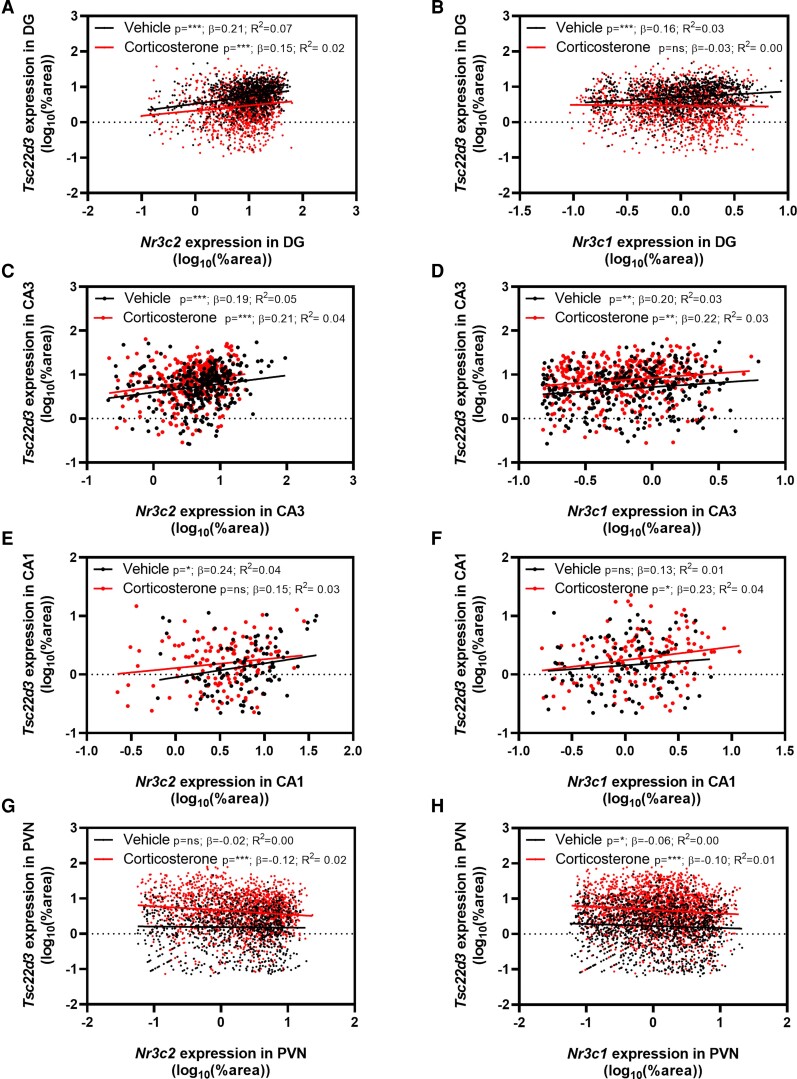
Mineralocorticoid receptor (MR) and glucocorticoid receptor (GR) messenger RNA (mRNA) levels poorly predict *Tsc22d3* expression in cells of the hippocampal and paraventricular nucleus of the hypothalamus (PVN). Cells from all mice were pooled per treatment for the correlation. Statistically significant but very weak correlations between *Nr3c2*, *Nr3c1*, and *Tsc22d3* were found both in vehicle- and corticosterone-treated animals in A to F, dentate gyrus (DG), CA3, CA1, and in G and H, PVN. All expression data were log10-transformed. Statistical significance was calculated with simple linear regression. The graphs show the significance (*P*), slope (β), and strength (*R*^2^) of the correlations found *P less than .05, **P less than .01, ***P less than .001.

As the results of the multiple regression showed that GR and MR mRNA expression, and not GR:MR ratio, can predict GILZ mRNA expression, we proceeded to explore whether GR and MR mRNA levels separately could predict GILZ mRNA at the cellular level. Single linear regression was performed on all cells of mice within the same treatment group. Significant, positive correlations between MR, GR, and GILZ mRNA expression were found in vehicle- and/or corticosterone-treated animals in various hippocampal subfields (Fig. 3A-3F). However, the correlations were generally very weak (*R*^2^ < 0.08), even when data were evaluated at the cellular level per mouse individually (data not shown). Similar results were obtained in the PVN (*R*^2^ < 0.03; Fig. 3G-3H). Therefore, we conclude that, despite some significant correlations, GR and MR mRNA levels poorly predict GILZ mRNA expression at the cellular level, as measured with the ISH approach that we used.

### Mineralocorticoid Receptor and Glucocorticoid Receptor Messenger RNA (mRNA) Levels Poorly Predict Glucocorticoid-induced Leucine Zipper mRNA Expression in Hippocampal Glutamatergic and GABAergic Neurons Under Basal Conditions

**Figure 4.**
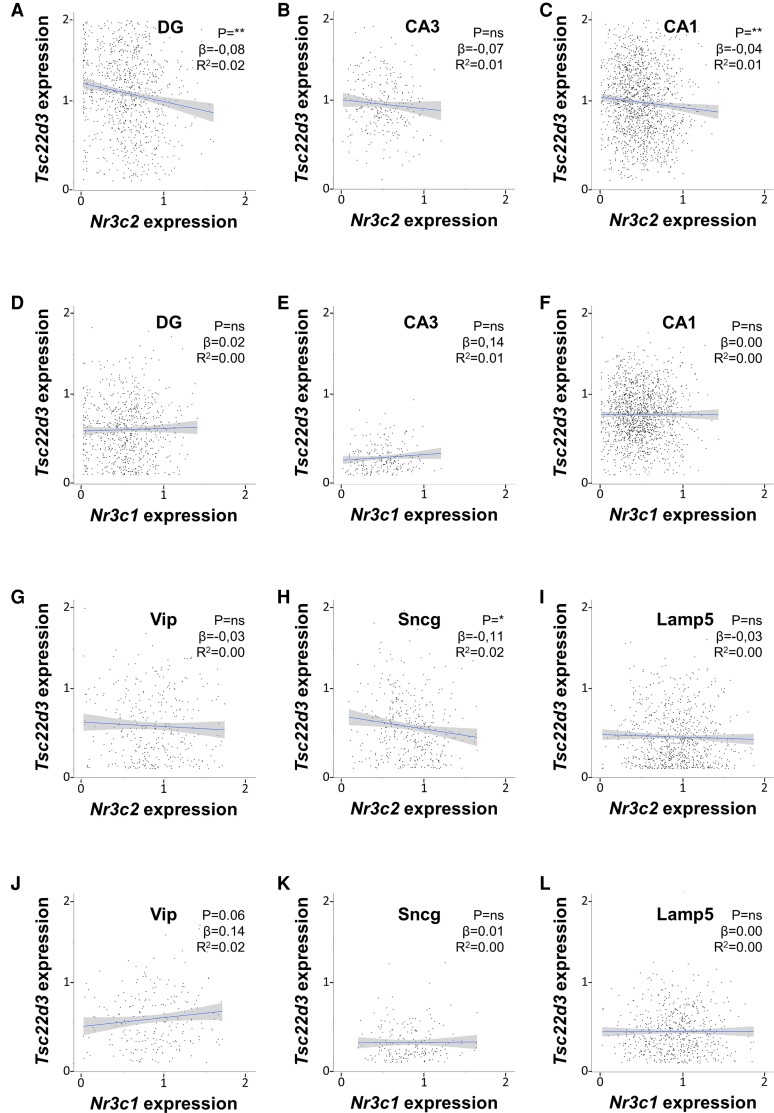
Mineralocorticoid receptor (MR) and glucocorticoid receptor (GR) messenger RNA (mRNA) levels do not predict glucocorticoid-induced leucine zipper (GILZ) mRNA expression in hippocampal glutamatergic and GABAergic neurons. Cellular MR (*Nr3c2*), GR (*Nr3c1*), and GILZ (*Tsc22d3*) mRNA levels were obtained from a previously published single-cell sequencing data set of naive mouse hippocampi. All expression values are log-normalized and scaled according to the Seurat pipeline. MR and GR mRNA levels poorly predicted *Tsc22d3* expression in A to F, glutamatergic neurons of the dentate gyrus (DG), CA3, and CA1 regions or in G to L, various subclasses of GABAergic neurons, that is, Vip, Sncg, and Lamp5 neurons. Data were analyzed with simple linear regression. The graphs show the 95% CI, as well as the statistical significance (*P*), slope (β), and strength (*R*^2^) of the correlations found. *P less than .05, **P less than .01.

The ISH data do not differentiate between neuronal cell types, and the weak correlations may additionally be due to the low sensitivity of the assay or undersampling. We therefore explored the association between corticosteroid receptors and GILZ mRNA expression using a previously published single-cell RNA sequencing data set of naive mouse hippocampi. In this data set, 4 glutamatergic and 5 GABAergic neuronal types could be identified. Glutamatergic neurons were distinguished based on the subclassification of hippocampal regions (CA regions and DG). The CA2 region was excluded from regression analyses because of low cell counts. In glutamatergic neurons, MR mRNA levels were significantly and negatively correlated with GILZ mRNA in the CA1 and the DG, but not in CA3 (Fig. 4A-4C). However, the strength of these correlations was again very weak (*R*^2^ < 0.03). No significant correlations were found between GR and GILZ mRNA levels in glutamatergic neurons (Fig. 4D-4F). From the 5 GABAergic neurons identified, the parvalbumin and somatostatin neurons were excluded from analyses because of low cell counts, leaving the lysosomal-associated membrane protein (Lamp5), synuclein gamma (Sncg), and vasoactive intestinal peptide (Vip) positive neurons. A significant, but very weak negative correlation was found between MR and GILZ mRNA in Sncg neurons, but not in Lamp5 and Vip neurons (*R*^2^ = 0.02; Fig. 4G-4I). GR mRNA levels did not correlate with GILZ mRNA in GABAergic neurons (Fig. 4J-4L). In conclusion, MR and GR mRNA levels poorly predicted GILZ mRNA expression in basal conditions both in glutamatergic and GABAergic neurons.

### Mineralocorticoid Receptor and Glucocorticoid Receptor Messenger RNA Levels do not Predict *Fkbp5* Expression in Hippocampal Glutamatergic and GABAergic Neurons

**Figure 5.**
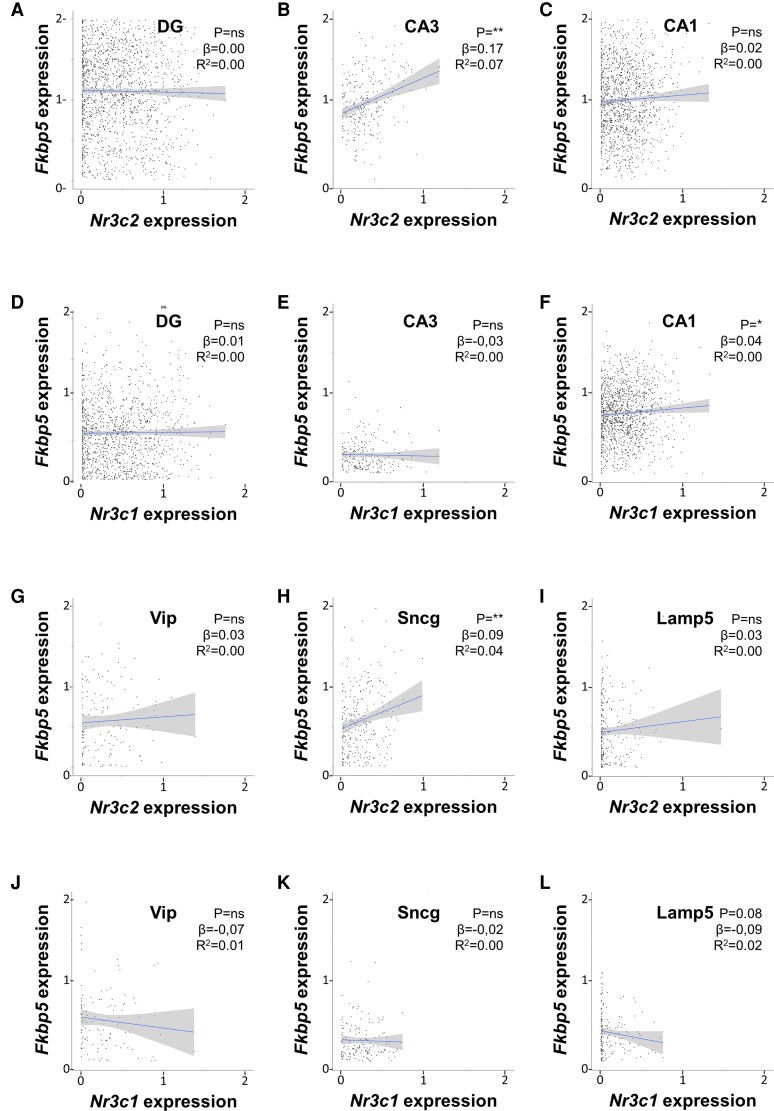
Mineralocorticoid receptor (MR) and glucocorticoid receptor (GR) messenger RNA (mRNA) levels do not predict *Fkbp5* expression in hippocampal glutamatergic and GABAergic neurons. Cellular MR (*Nr3c2*), GR (*Nr3c1*), and glucocorticoid-induced leucine zipper (GILZ) (*Tsc22d3*) mRNA levels were obtained from a previously published single-cell sequencing data set of naive mouse hippocampi. All expression values are log-normalized and scaled according to the Seurat pipeline. MR and GR mRNA levels poorly predicted *Fkbp5* expression in A to F, glutamatergic neurons of the dentate gyrus (DG), CA3, and CA1 regions, or in G to L, various subclasses of GABAergic neurons, that is, Vip, Sncg, and Lamp5 neurons. Data were analyzed with simple linear regression. The graphs show the 95% CI, as well as the statistical significance (*P*), slope (β), and strength (*R*^2^) of the correlations found. *P less than .05, **P less than .01.

To assess the generality of our findings, we extended our analysis in the single-cell sequencing data set to another shared GR and MR target gene: FK506 binding protein 5 (*Fkbp5*). Using the same single-cell RNA sequencing data set, GR and MR mRNA levels were correlated with *Fkbp5* expression in hippocampal glutamatergic and GABAergic neurons. A statistically significant but very weak correlation was found between MR and FKBP5 mRNA levels in glutamatergic neurons of the CA3 region, but not in the DG or CA1 (*R*^2^ = 0.07; Fig. 5A-5C). GR mRNA levels did not significantly predict *Fkbp5* expression in glutamatergic neurons (Fig. 5D-5F). Only in GABAergic Sncg neurons did MR mRNA levels significantly but weakly predict *Fkbp5* expression (*R*^2^ = 0.04; Fig. 5G-5I). GR mRNA levels were not associated with *Fkbp5* in hippocampal GABAergic neurons (Fig. 5J-5L). To conclude, similarly to *Tsc22d3*, *Fkbp5* expression could not robustly be predicted by corticosteroid receptor expression in hippocampal glutamatergic and GABAergic neurons.

### *11βHsd1* Expression Weakly Correlates With Glucocorticoid-induced Leucine Zipper and FKBP5 Messenger RNA Expression in Hippocampal Glutamatergic and GABAergic Neurons

**Figure 6.**
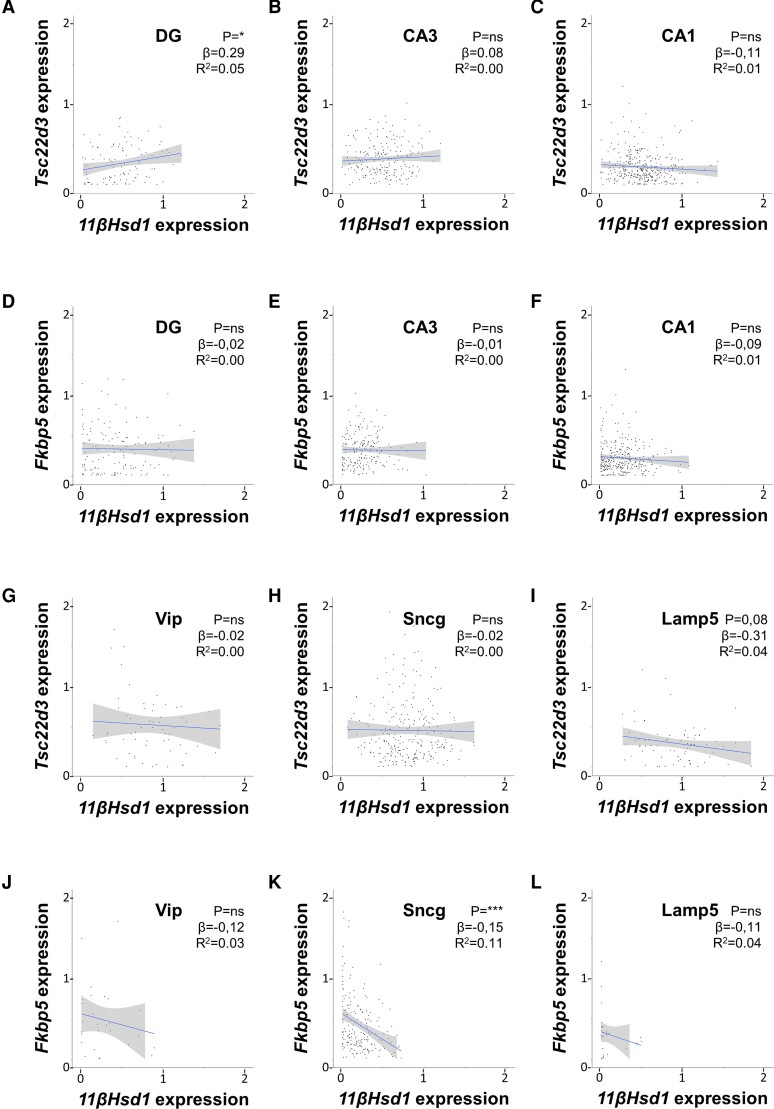
*11βHsd1* expression weakly correlates with glucocorticoid-induced leucine zipper (GILZ) or FKBP5 messenger RNA (mRNA) expression in hippocampal glutamatergic and GABAergic neurons. Cellular 11βHSD1, GILZ, and FKBP5 mRNA levels were obtained from a previously published single-cell sequencing data set of naive mouse hippocampi. All expression values are log-normalized and scaled according to the Seurat pipeline. A to F, *11βHsd1* expression poorly predicted *Tsc22d3* or *Fkbp5* expression in A to F, glutamatergic neurons of the dentate gyrus (DG), CA3, and CA1 regions, or in G to L, various subclasses of GABAergic neurons, that is, Vip, Sncg, and Lamp5 neurons. Data were analyzed with simple linear regression. The graphs show the 95% CI, as well as the statistical significance (*P*), slope (β), and strength (*R*^2^) of the correlations found. *P less than .05, ***P less than .001.

Next to receptor levels, ligand levels importantly determine the transcriptional activity of corticosteroid receptors. The hypothalamic-pituitary-adrenal axis controls circulating glucocorticoid hormone levels, while local variations in hormone levels are caused by activity of the 11β-hydroxysteroid dehydrogenase type 1 (*11βHsd1*) enzyme, which converts glucocorticoids from their inactive to their active form [33]. Therefore, we investigated how *11βHsd1* expression relates to GILZ and FKBP5 mRNA levels at the single-cell level. In glutamatergic neurons, 11βHsd1 mRNA did not predict GILZ and FKBP5 mRNA expression (*R*^2^ < 0.06; Fig. 6A-6F). No statistically significant correlations were found between 11βHSD1 and GILZ mRNA in GABAergic neurons (Fig. 6G-6I). A significant but weak correlation was found between *11βHsd1* and *Fkbp5* expression in Sncg neurons (*R*^2^ = 0.11; Fig. 6J-6L). Taken together, *11βHsd1* expression did not explain target gene expression in either glutamatergic or GABAergic neurons.

## Discussion

This study explored how GR and MR mRNA levels and GR:MR balance predict receptor activity, as measured by mRNA expression of target genes GILZ and FKBP5. At the tissue level, MR mRNA levels were positively correlated with GILZ mRNA under vehicle conditions in the CA1 and CA3, and under corticosterone conditions in the DG and PVN. In addition, MR mRNA levels were consistently higher in GILZ*-*positive cells. GR mRNA levels did not correlate with GILZ mRNA expression, except in the CA1 region of corticosterone-treated animals. The effect of the GR:MR ratio on GILZ mRNA expression was a net sum of the individual effects of GR and MR. However, we observed that the presence of MR in GR-positive cells may reduce the *Tsc22d3* induction on corticosterone. At the cellular level, MR and GR mRNA levels generally poorly predicted GILZ mRNA levels, both in data generated by ISH and single-cell RNA sequencing. Similarly, MR and GR mRNA levels were not associated with *Fkbp5* expression at the single-cell level. Taken together, our data show that depending on the hippocampal subfield and corticosterone concentrations, MR (but not GR) mRNA levels can limit the mRNA expression of GILZ in the hippocampus and PVN at the tissue level, but not at the cellular level.

In line with the notion that MR activity is controlled by the relative abundance of receptors due to the high affinity for corticosterone and its occupancy under basal conditions, we observed that MR mRNA levels limit GILZ mRNA expression at the tissue level. It differed per hippocampal subfield whether MR was limiting under low- or high-ligand conditions. This may be due to region-specific translocation dynamics, which were previously shown to differ between the CA1 and DG, although these previous observations do not directly explain our findings [34]. There may also be regional differences in posttranslational modifications or expressed splice variants of MR, which both influence the sensitivity and transcriptional activity of the receptor [35-37]. Glucocorticoid levels, and thus receptor occupancy, can additionally differ per subfield due to the activity of glucocorticoid-amplifying enzyme 11βHSD1. In single-cell RNA sequencing data, *11βHsd1* expression did not predict GILZ mRNA expression at the basal level, but mRNA levels do not necessarily reflect enzymatic activity. Therefore, it is possible that local 11βHSD1 activity was higher in regions where MR mRNA levels limited target gene expression in vehicle-treated animals [38-40]. Supporting that ligand levels limit GR action, GR did not limit GILZ mRNA expression in most investigated regions including CA3, where overall GR expression is low. Since GR was limiting in a region where GR is highly expressed, this again underlines the importance of other cell- or region-specific factors that, next to absolute receptor levels, eventually determine GR-mediated transcription.

We did not find a transcriptional basis for the U-shaped response to glucocorticoids that is functionally observed. The transcriptional changes underlying such a relationship perhaps reflect regulation of MR- and GR-specific target genes, which would code for proteins with opposite actions [2]. This is also suggested by chromatin immunoprecipitation–sequencing studies that show that GRE binding is the dominant mode of signaling in the rodent hippocampus, arguing against GR-mediated transrepression through tethering to other transcription factors [3, 41, 42]. While a U-shaped response to hormones has been described in the CA1, a bell-shaped and linear relationship has been described for the DG and PVN, respectively [8, 9]. Since we did not find differences in the transcriptional regulation of GILZ between these regions, this further supports that the opposite actions of MR and GR involve separate target genes.

Despite the strong correlations at the tissue level, GR and MR mRNA levels poorly predicted GILZ or FKBP5 mRNA expression at the cellular level. The lack of correlation between MR and FKBP5 mRNA at the cellular level is remarkable in light of the recently reported role of MR to drive basal FKBP5 expression [22]. However, in that study the relation between MR and FKBP5 was not studied at the single-cell level. It is possible that the strong tissue correlations in our study reflected an association between receptors and target genes expressed in different cells. In that case, these correlations may not reflect causality between receptor levels and target gene expression within the same cell. This is not very likely, however, as most cells expressed both receptors and GILZ mRNA. In addition, the enrichment of MR in cells expressing GILZ, as well as the observation that GILZ mRNA levels were higher when MR was present in GR-expressing cells, suggest that MR could even be a driver of basal GILZ mRNA expression. The lack of effect at the cellular level may rather reflect a sensitivity issue of the ISH technique used or undersampling. Also sequencing depth per cell in the single-cell RNA sequencing study may not have been sufficient for our purposes. For the ISH technique, different cell sizes may have introduced considerable variation in the percentage area readout parameter. Furthermore, the directionality of the correlations between receptors and target genes may differ per cell type (positive/negative/no effect), which could have reduced the overall effect. This effect dilution is especially observed in the highly heterogeneous cell population of the PVN, where statistical significance at the mouse level (*R*^2^ = 0.81) became negligible (*R*^2^ = 0.02) at the cellular level. However, when we differentiated between glutamatergic and GABAergic neurons in a single-cell RNA sequencing data set, we did not find stronger correlations or major differences in directionality between these 2 cell types.

Despite the weak correlations found both with ISH and single-cell RNA sequencing, the directionality of the correlations found markedly differed between the 2 techniques. For example, with ISH MR and GILZ mRNA levels correlated positively in CA1 and DG cells under vehicle conditions, whereas this correlation was negative in the glutamatergic and GABAergic cell populations in these areas. As explained earlier, cell specificity may play a role here, as the ISH data and not the single-cell RNA sequencing data included nonneuronal cells, which express GILZ mRNA more frequently compared to other cell populations in the hippocampus [43]. Alternatively, GRs and MRs may have been more occupied with corticosterone in the ISH data set because mice were vehicle-injected before being killed, whereas mice were naive in the single-cell sequencing experiment [28]. Finally, the discrepancy may have also arisen from sex-specific glucocorticoid signaling, as male mice were used for the ISH experiment, whereas both sexes were used in the single-cell sequencing experiment [28]. This crosstalk may even be cell specific, as mainly in neuronal cells a high colocalization was found between the corticosteroid receptors and the androgen and progesterone receptors at the mRNA level [43].

In this study we chose to measure mRNA levels of corticosteroid receptors as these are often used to assess receptor status (eg, [19, 20, 44]). However, it is likely that parameters other than mRNA levels can better approximate actual receptor activity. Since mRNA levels do not always reflect protein status, protein levels may better reflect the “capacity” of the receptor [45, 46]. In addition, the transcriptional activation of genes is determined by many other factors, including the bioavailability of the ligand, on/off binding kinetics of the receptors at the genome, posttranslational modifications of the receptors, and the exact protein composition of the receptor-ligand transcription complex [47-49]. Therefore, it is likely that multiple variables need to be included in the regression model to robustly predict target gene expression at the cellular level, although it is also likely there will remain some degree of randomness, given the stochastic events that occur on receptor-promotor binding that further cause intracellular variability in the transcriptional response [50].

Ultimately, we showed that MR mRNA levels, and GR mRNA levels to a smaller extent, could predict GILZ mRNA expression at the tissue level, but not at the cellular level. While we cannot fully exclude that GR and MR mRNA may predict other GR/MR target genes, we recommend caution when interpreting mRNA levels of corticosteroid receptors and the GR:MR balance to assess the response to hormones in future studies, as many other cell- and region-specific factors determine the glucocorticoid-driven transcriptional outcome.

## Data Availability

Some or all data sets generated during and/or analyzed during the present study are not publicly available but are available from the corresponding author on reasonable request.

## Abbreviations

*11βHsd1*: 11β-hydroxysteroid dehydrogenase type 1
DAPI, 4': 6-diamidino-2-phenylindole
DG: dentate gyrus
GILZ: glucocorticoid-induced leucine zipper
GR: glucocorticoid receptor
GRE: glucocorticoid response element
ISH: in situ hybridization
Lamp5: lysosomal-associated membrane protein
MR: mineralocorticoid receptor
mRNA: messenger RNA
PVN: paraventricular nucleus of the hypothalamus
Sncg: synuclein gamma
Vip: vasoactive intestinal peptide
